# An *E. coli* biosensor for screening of cDNA libraries for isochorismate pyruvate lyase-encoding cDNAs

**DOI:** 10.1007/s00438-018-1450-5

**Published:** 2018-05-23

**Authors:** Yingjie Zhou, Johan Memelink, Huub J. M. Linthorst

**Affiliations:** Institute of Biology, Leiden University, P.O. Box 9505, 2300 RA Leiden, The Netherlands

**Keywords:** Salicylic acid, Isochorismate pyruvate lyase, Screening, *Arabidopsis thaliana*

## Abstract

Salicylic acid (SA) is an essential hormone for development and induced defense against biotrophic pathogens in plants. The formation of SA mainly derives from chorismate via demonstrated isochorismate synthase (ICS) and presumed isochorismate pyruvate lyase (IPL)-mediated steps in *Arabidopsis thaliana*, but so far no plant enzyme displaying IPL activity has been identified. Here, we developed an *E. coli* SA biosensor to screen for IPL activity based on the SalR regulator/*salA* promoter combination from *Acinetobacter* sp ADP1, to control the expression of the reporter *luxCDABE*. The biosensor was responsive to micromolar concentrations of exogenous SA, and to endogenous SA produced after transformation with a plasmid permitting IPTG-inducible expression of bacterial IPL in this biosensor strain. After screening a cDNA library constructed from turnip crinkle virus (TCV)-infected Arabidopsis ecotype Di-17, we identified an enzyme, PRXR1, as a putative IPL that converts isochorismate into SA. Our results provide a new experimental approach to identify IPL and new insights into the SA biosynthesis pathway in Arabidopsis.

**Electronic supplementary material:**

The online version of this article (10.1007/s00438-018-1450-5) contains supplementary material, which is available to authorized users.

## Introduction

Salicylic acid (SA) is a small phenolic compound present in plants and bacteria. In plants, SA is an essential signaling molecule that mediates defense against infections with biotrophic pathogens and is produced to regulate several physiological functions, including flowering induction and seed germination (Rivas-San Vicente and Plasencia 2011; Spoel and Dong 2012). SA functions as a precursor of siderophores in many bacteria, such as pyochelin in *Pseudomonas aeruginosa*, yersiniabactin in *Yersinia pestis* and *Y. enterocolitica*, and mycobactin in *Mycobacterium tuberculosis* (Cox et al. 1981; Crosa and Walsh 2002; Gaille et al. 2003; Pelludat and Brem 2003). The SA biosynthesis pathway in bacteria is well known. Chorismate, the end product of the shikimate pathway, is converted to SA by two enzymes: isochorismate synthase (ICS) and isochorismate pyruvate lyase (IPL), encoded in *P. aeruginosa* by the genes *pchA* and *pchB*, and in *P. fluorescens* by the genes *pmsA* and *pmsB* (Mercado-Blanco et al. 2001; Gaille et al. 2002). In *Y. enterocolitica* and *M. tuberculosis*, salicylate synthase (Irp9 and MbtI, respectively) carries out the direct conversion of chorismate to salicylate (Kerbarh et al. 2005; Zwahlen et al. 2007). SA biosynthesis in plants also originates from chorismate, but here two different pathways have been suggested. However, neither of these branches has been conclusively elaborated (Dempsey et al. 2011; Boatwright and Pajerowska-Mukhtar 2013; Dempsey and Klessig 2017).

The first branch involves the enzyme phenylalanine ammonia-lyase (PAL) which converts phenylalanine to cinnamic acid. The second pathway is dependent on ICS, which catalyzes the conversion of chorismate to isochorismate (Wildermuth et al. 2001; Strawn et al. 2007; Garcion et al. 2008). The reaction leading to SA is expected to involve an as of yet unidentified IPL enzyme (Macaulay et al. 2017). Due to the chloroplast localization of ICS in plants and the fact that transgenic Arabidopsis overexpressing the bacterial *NahG* (salicylate hydroxylase) gene in the chloroplasts failed to accumulate SA after pathogen infection or UV exposure (a stimulus for SA accumulation), this pathway is likely to be localized in the chloroplasts (Nawrath et al. 2002; Garcion et al. 2008; Fragnire et al. 2011). The contribution of the isochorismate pathway for SA biosynthesis differs depending on the plant species. For instance, the isochorismate biosynthesis pathway contributes more than 90% of the SA produced during pathogenesis in Arabidopsis, while the PAL and IC pathways are equally important for pathogen-induced SA biosynthesis in soybean (Garcion et al. 2008; Shine et al. 2016).

Conventional methods of SA quantification in plant tissues are based on high-performance liquid chromatography (HPLC) or gas chromatography/mass spectrometry (GC/MS), which required time-consuming extraction steps with organic solvents (Nawrath and Métraux 1999; Verberne et al. 2000; Huang et al. 2006; Garcion et al. 2008). Given the importance of SA, salicylate-responsive biosensors were designed to detect SA and SA derivatives faster and for larger scale sample ranges (Huang et al. 2005; Shin 2010). Huang et al. (2005) engineered an *Acinetobacter* biosensor originating from *Acinetobacter baylyi* sp. ADP1. This strain is able to use SA as sole carbon source (Jones et al. 2000). It contains the *salA* operon encoding salicylate hydroxylase involved in SA degradation to catechol. Transcription of this operon is activated in the presence of SA that interacts with the regulatory protein SalR. SalR belongs to the LysR-type transcriptional regulator (LTTR) family of proteins that contain two typical major domains, i.e. an N-terminal helix–turn–helix motif (residues 18–47) and a C-terminal co-factor binding domain. SalR activates the transcription of *salA* though binding to a dyadic sequence (TTCA-N_12_-TGAT) around − 190 bp upstream of the *salA* transcription start site, in response to the inducer SA (Schell 1993; Jones et al. 2000). For the construction of the *Acinetobacter* biosensor, the *luxCDABE* operon from *Photorhabdus luminescens*, encoding luciferase and enzymes involved in biosynthesis of its substrate (Winson et al. 1998a, b) was integrated into the *Acinetobacter* genome, resulting in *Acinetobacter* strain ADPWH*_lux*. This biosensor is highly sensitive to SA, methyl-SA, and acetyl-SA, and has been used to monitor SA in plant tissues (Huang et al. 2006; DeFraia et al. 2008; Ding et al. 2014).

Large-scale genetic screens for mutants in Arabidopsis with reduced SA accumulation after pathogen infection have resulted in identification of the gene *EDS5* encoding a chloroplast-localized MATE (multidrug and toxin extrusion) transporter shown to transport SA from the chloroplast to the cytosol, and in the identification of the gene encoding the SA biosynthetic enzyme ICS1 (Nawrath and Métraux 1999; Dewdney et al. 2000; Serrano et al. 2013). For years, biologists have looked for an enzyme with IPL activity in plants to complete the ICS branch in the SA biosynthesis pathway, but as of yet no such activity was found.

In this article, we describe the construction and characterization of a plasmid-based *E. coli* SA biosensor to screen a cDNA library of Arabidopsis for genes encoding the unidentified IPL. The sensing components, including the SA-inducible promoter P*salA*, the reporter operon *luxCDABE* and the *salR* gene encoding the LysR-type regulator of *salA*, were subcloned from *Acinetobacter* ADPWH*_lux* into a plasmid vector. As proof of principle, an expression plasmid was constructed carrying the *pmsB* gene from *P. fluorescens* encoding bacterial IPL behind the IPTG-inducible *lac* promoter, which served as a positive control for the biosensor. Since *E. coli* contains the *entC* gene encoding ICS that supports the production of isochorismate, transformation with the *pmsB* plasmid resulted in synthesis of SA, which activated the SalR regulator, leading to expression of the *lux* genes and generation of light. This made it possible to use the *E. coli* biosensor strain to screen a cDNA expression library for cDNAs encoding proteins with IPL activity, by determining the light production of individual clones. Based on this approach, we identified PRXR1 as a potential IPL in Arabidopsis.

## Results

### Construction of a luminescence reporter plasmid

**Fig. 1:**
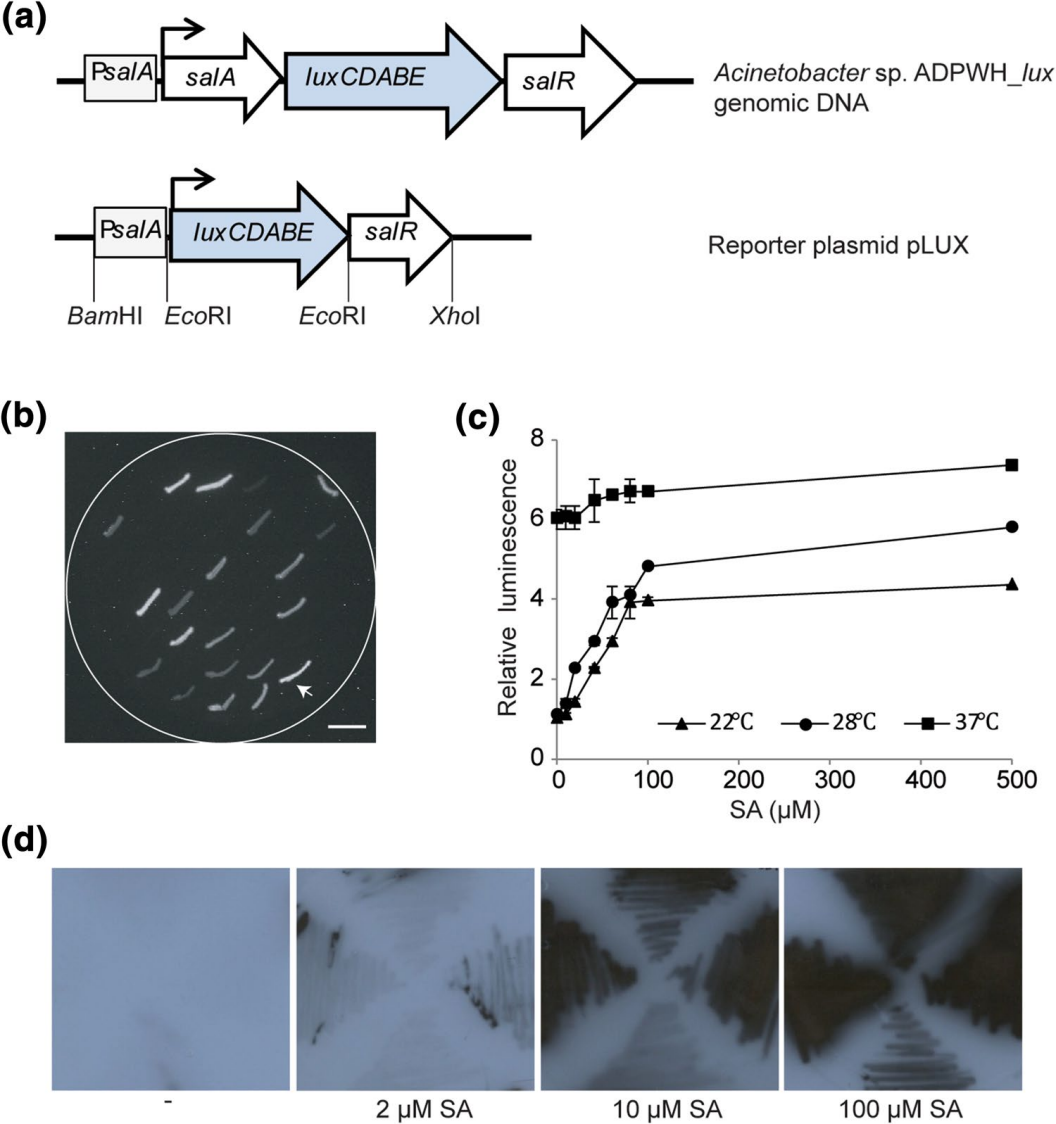
*E. coli*-based whole-cell biosensor XL1-LUX induction by SA. **a** Representation of chromosomally integrated *luxCDABE* in *Acinetobacter* sp. ADPWH_*lux* (Huang et al. 2005) and pLUX reporter cassette. **b** Selection of *E. coli* XL1-LUX transformants on LC plate supplemented with SA. The *E*. coli transformant selected for our experiments is indicated by the arrow. Scale bar = 1 cm. **c** Relative luminescence values from XL1-LUX grown in liquid medium with different concentrations of SA, incubated at different temperatures. Luminescence values of uninduced cells (0 µM SA) incubated at room temperature (22 °C) were taken as 1. Error bars represent the mean ± SE (*n* = 3). **d** Luminescence images on X-ray film of XL1-LUX streaked onto solid medium containing different concentrations of SA

Our aim was to engineer a biosensor that could detect endogenous SA in *E. coli*. In our construct, the promoterless *luxCDABE* operon encoding luciferase was cloned behind the upstream promoter region of the *salA* operon (P*salA*) to replace the original *salA* gene. SA-inducible expression of the resulting promoter-reporter operon is regulated by the product of the *salR* gene (Jones et al. 2000; Mercado-Blanco et al. 2001) (Fig. 1a). The resulting plasmid containing *salR* downstream of the newly created P*salA::luxCDABE* operon was named pLUX. *E. coli* XL-1 Blue MRF’ cells carrying pLUX (XL1-LUX) were able to produce luminescence due to the constitutive expression of *luxCDABE* in the presence of exogenous SA and this phenomenon could be easily captured by auto-exposure in the dark (Fig. 1b).

In general, XL1-LUX exhibited increased luciferase expression for concentrations of salicylate between 0 and 500 µM when the cells were grown in liquid medium at room temperature (22 °C) and 28 °C with fourfold and fivefold increased luminescence compared to uninduced cells (Fig. 1c). When incubated at 37 °C, uninduced cells (0 µM SA) had strong basal luminescence that barely changed with higher SA concentrations. When grown on solid medium at room temperature, luminescence responded likewise to increasing SA concentrations (Fig. 1d). These results indicated that the XL1-LUX biosensor was capable of detecting exogenous SA with a useful dose–response relationship at lower growth temperatures.

### Endogenous SA generation and luminescence monitoring

**Fig. 2:**
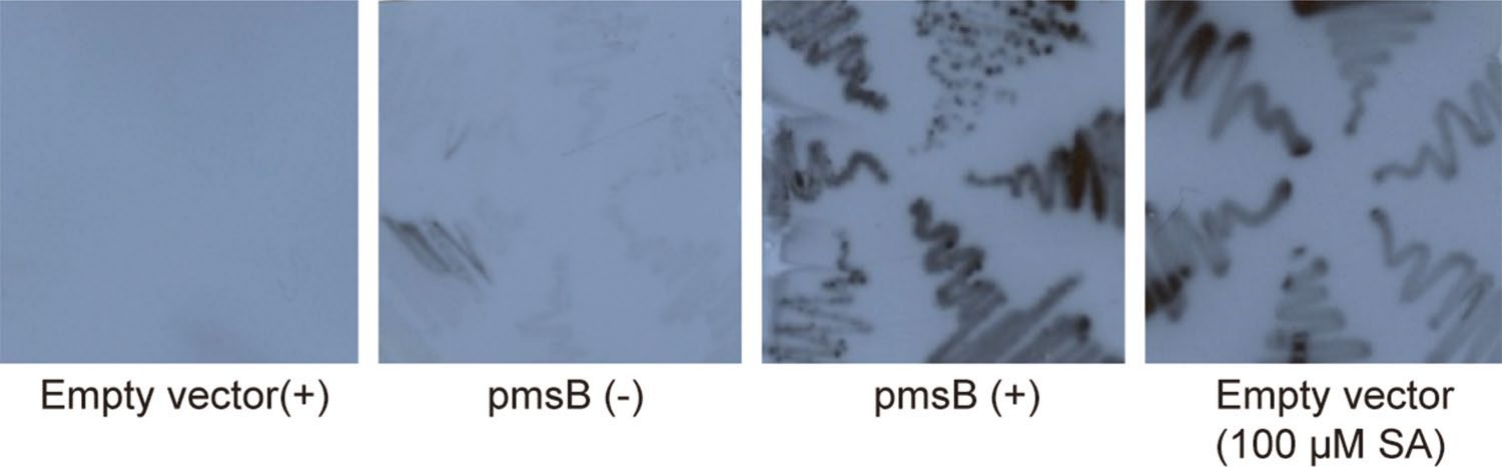
*E. coli*-based biosensor XL1-LUX monitoring endogenous SA. Images of luminescence of XL1-LUX containing empty pBK-CMV or pBK-CMV-pmsB in the absence (−) or presence of IPTG (+). Luminescence of XL1-LUX with empty vector in the presence of 100 µM SA was included as a control

In *E. coli*, isochorismate synthase EntC transforms chorismate into isochorismate, which provides substrate for enterobactin biosynthesis (Ozenberger et al. 1989; Walsh et al. 1990). PmsB, originally from *P. fluorescens*, can convert the endogenous isochorismate into SA when expressed in *E. coli* (Mercado-Blanco et al. 2001; Zhou 2018). In order to test whether XL1-LUX is able to respond to endogenous SA, a pBK-CMV plasmid derivative carrying the *pmsB* gene under the control of the strong isopropyl thio-β-D-galactoside (IPTG)-inducible lac promoter (pBK-CMV-pmsB), was transformed into XL1-LUX (Fig. 2). As a negative control, a pBK-CMV vector without insertion was transformed into XL1-LUX. We observed stronger luminescence in the cells containing the expressed PmsB compared to the negative control and the uninduced cells (Fig. 2). This result indicated that *E. coli* XL1-LUX was sensitive to the endogenously produced SA.

### Arabidopsis cDNA library screening using XL1-LUX

**Fig. 3:**
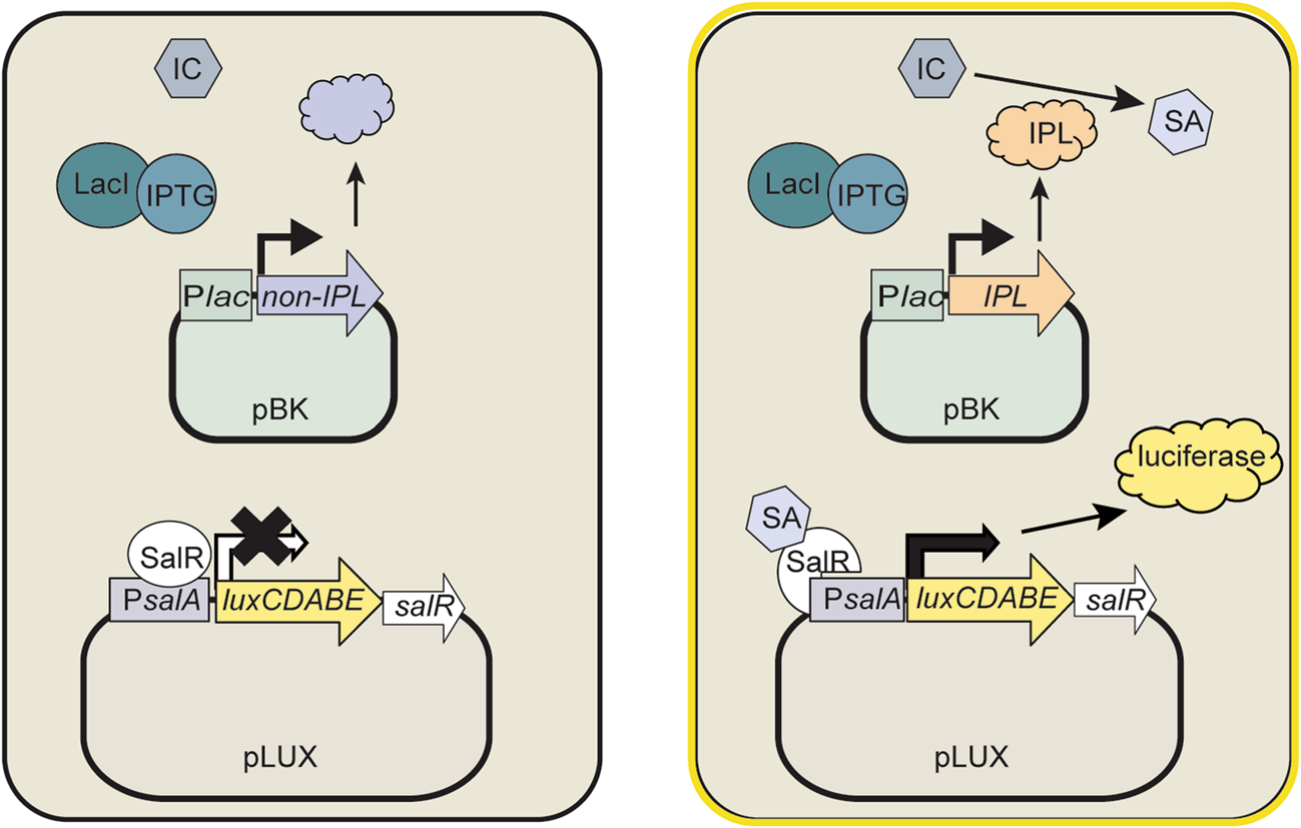
Schematic representation of cDNA library screening in XL1-LUX. *E. coli* cells contain the reporter plasmid pLUX and a cDNA-harboring pBK-CMV plasmid. In the presence of IPTG, cells containing a cDNA encoding an enzyme with IPL activity are able to convert endogenous isochorismate to SA, resulting in activation of salR and accumulation of luciferase and its substrates that lead to light production

Previous work showed that inoculation of turnip crinkle virus (TCV) on the resistant Arabidopsis ecotype Dijon (Di-17) results in an increase in SA content, in the development of a hypersensitive response (HR) and in induction of *PR* gene expression (Uknes et al. 1993; Dempsey et al. 1997; Kachroo et al. 2000; Chandra-Shekara et al. 2006). This indicates that the genes involved in SA biosynthesis are active upon TCV inoculation. To find genes encoding IPL in Arabidopsis using the biosensor, a cDNA plasmid (pBK-CMV) library made from mRNA of Arabidopsis Di-17 leaves infected by TCV (Ausubel et al. 1987; Cooley et al. 2000) was transformed into *E. coli* XL1-LUX. Cells were selected for the presence of both the pLUX and the cDNA plasmid using carbenicillin and kanamycin, respectively (Fig. 3).

**Fig. 4:**
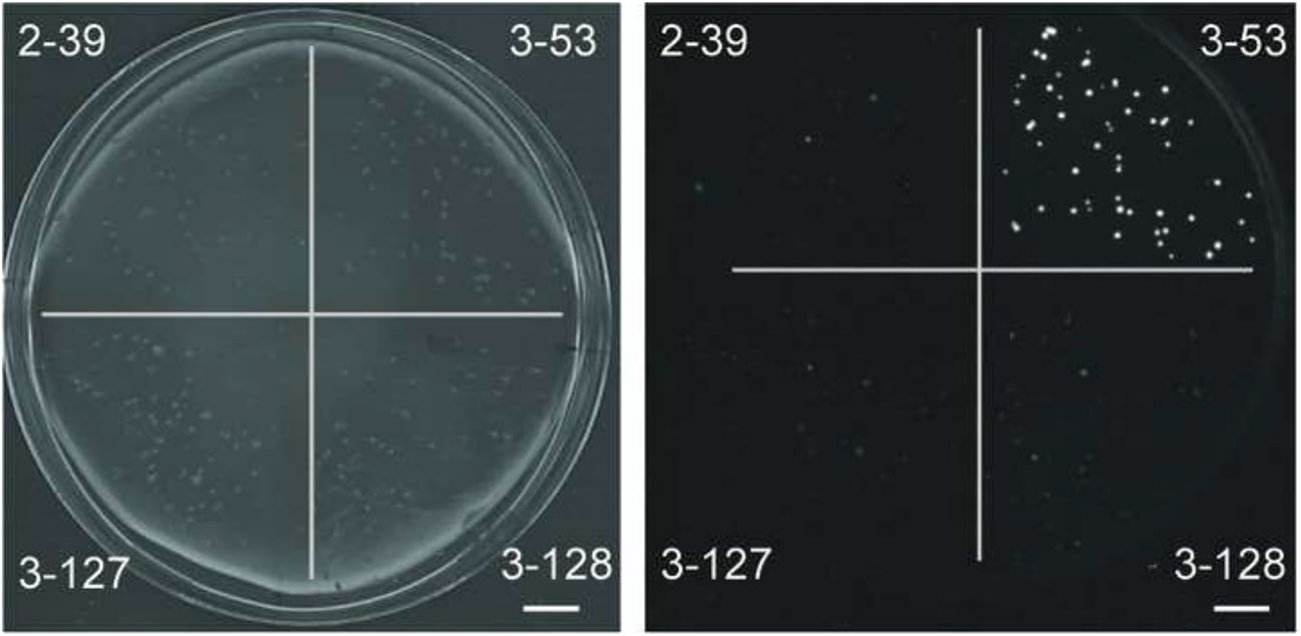
Luminescence scanner image of a Petri dish containing *E. coli* colonies from the cDNA library screening. The plate is divided in quadrants with colonies obtained after retransformation of XL1-LUX with four different plasmids (indicated by numbers) picked up in the screening, photographed in the light (left) or dark (right). Scale bar = 1 cm

One and a half million *E. coli* transformants were screened, resulting in approximately 1000 colonies that were able to generate higher luminescence than the background. Those colonies were recovered and streaked onto fresh medium. From 220 colonies that were selected, the pBK-CMV plasmids were isolated and re-transformed into XL1-LUX. This resulted in 43 clones displaying relatively strong luminescence (Fig. 4). The cDNAs from these clones were sequenced and 12 of them were in frame with the β-galactosidase α-fragment (Table S1).

### SA accumulation in *E. coli* expressing candidate cDNAs

**Fig. 5:**
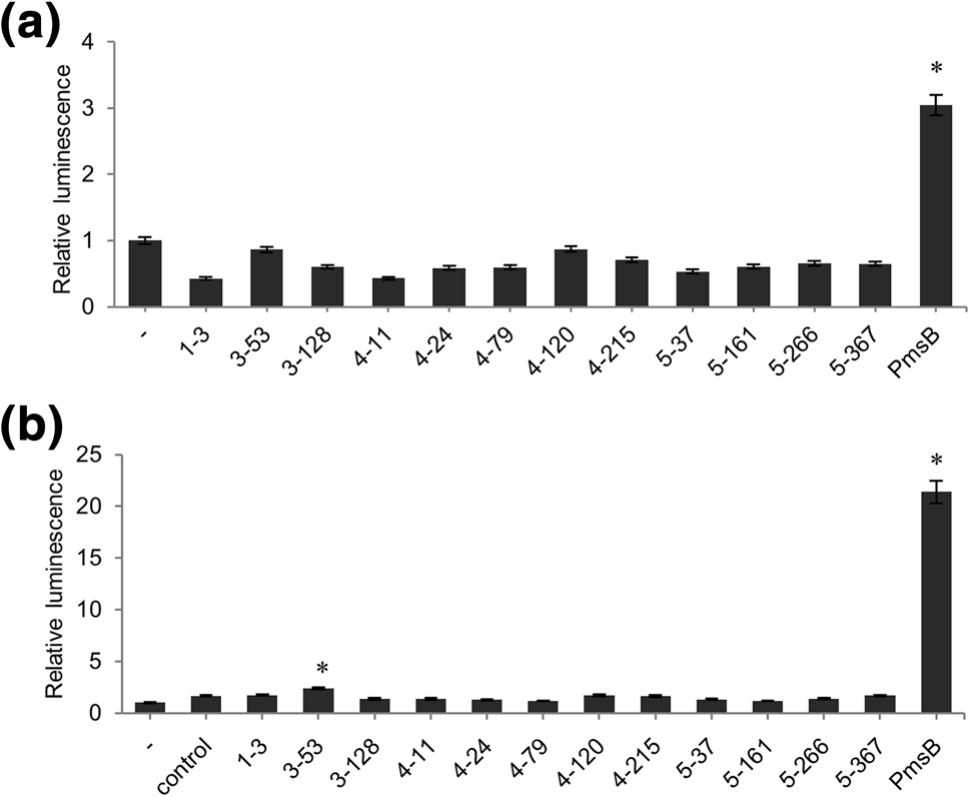
SA production in crude *E. coli* cell extracts, as measured through the *Acinetobacter* biosensor. **a** Luminescence values obtained with crude *E. coli* cell extracts from IPTG-induced XL1-Blue MRF’ containing indicated pBK-CMV plasmids from the screening. “-” indicates uninduced *Acinetobacter* cells. The asterisk indicates a significant difference in comparison to the uninduced *Acinetobacter* cells (*P* < 0.05). **b** Luminescence values obtained with crude *E. coli* cell extracts from IPTG-induced BL21 (DE3) pLysS transformed with the indicated plasmids, crude cell extract from *EntC*-overexpressing *E. coli* and incubated for 1 h with chorismate. “-” indicates non-induced *Acinetobacter* cells. “control” indicates *Acinetobacter* cells that were induced by a mixture of crude cell extract from *entC*-overexpressing *E. coli* and chorismate. Asterisks indicate significant differences in comparison to the control (*P* < 0.05). Luminescence values of uninduced *Acinetobacter* cells were taken as 1. Luminescence values were converted to SA concentrations using a standard curve similar to the one shown in Fig. 6b. Error bars represent the mean ± SE (*n* = 3)

To determine if the candidate cDNAs can direct SA accumulation in *E. coli*, we transformed the corresponding pBK-CMV plasmids into XL1-Blue MRF’ and BL21 (DE3) pLysS, a strain that is optimized for high expression of cloned genes (Fig. 5). The *pmsB* gene cloned in pBK-CMV and pASK-IBA45plus served as positive controls. The positive control extract obtained from XL1-Blue MRF’ containing the *pmsB* gene showed slightly higher luminescence activity than the uninduced control. The crude cell extracts of the candidate strains had no effect on luminescence, suggesting that SA was not produced or produced in such low amounts in these cells that it was not detectable by *Acinetobacter* (Fig. 5a).

**Fig. 6:**
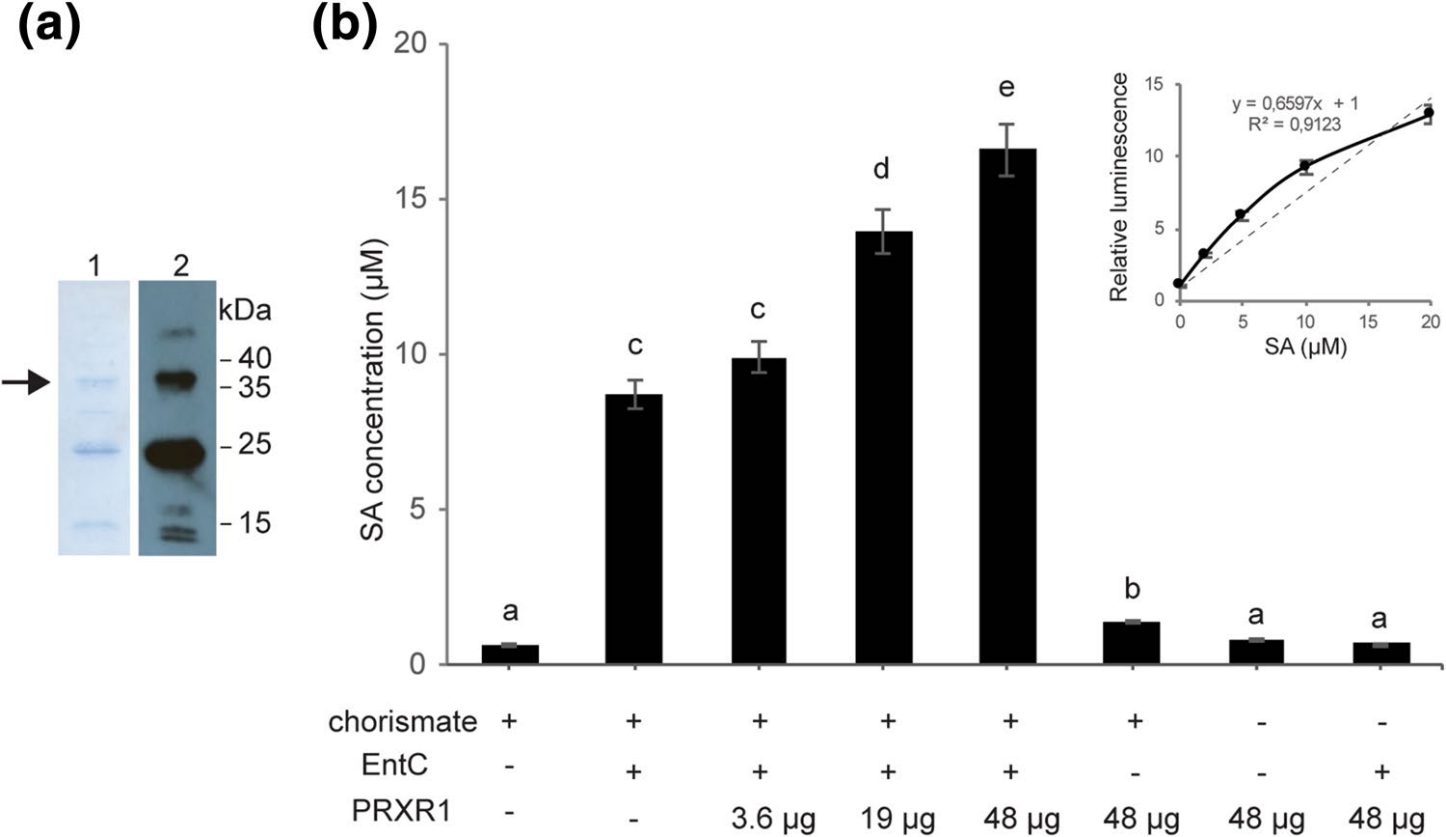
SA production in vitro with recombinant His-tagged PRXR1. **a** His-tagged PRXR1 protein was expressed in *E. coli*, purified and analyzed using SDS-PAGE and either stained with Coomassie Brilliant Blue (lane 1) or visualized with Western blotting using anti-His antibodies (lane 2). The positions of molecular mass markers are indicated in kDa. The arrow indicates the position of the recombinant PRXR1 protein band. **b** Production of SA in vitro, as measured with the *Acinetobacter* biosensor. Letters indicate significantly different groups (*P* < 0.05). Error bars represent the mean ± SE (*n* = 3). This experiment was repeated three times with similar results. The inset shows the calibration curve used for converting luminescence values to SA concentrations

We reasoned that *E. coli* XL1-Blue MRF’ might not produce enough isochorismate to allow sufficiently high production of SA, or that expression of the cDNAs was not sufficiently high in these cells. When crude cell extracts of these cells were incubated with chorismate, together with a crude cell extract of BL21 (DE3) pLysS transformed with an overexpression construct of the bacterial *entC* gene encoding isochorismate synthase, the SA production by the control pmsB extract was considerably higher. Of the candidate clones, only clone 3–53 resulted in low, but reproducible levels of SA. Sequencing revealed that the cDNA of plasmid 3–53 encodes a peroxidase named PRXR1 (also called ATP1a) (Fig. 5b).

### IPL activity of PRXR1

To establish whether PRXR1 catalyzes the conversion of isochorismate into SA, recombinant His-tagged PRXR1 protein was expressed in *E. coli* and purified by Ni–NTA agarose chromatography. The molecular subunit mass of His-PRXR1 protein was evaluated by 15% SDS-PAGE followed by Coomassie Brilliant Blue staining or by Western blot hybridization using anti-His antibodies. As shown in Fig. 6a, a band was visible at the expected position of 38 kDa for the recombinant His-tag-PRXR1 protein. All Coomassie Brilliant Blue-stained bands showed up on the Western blot, indicating that all proteins in the Ni–NTA purified sample were His-tagged PRXR1 and degradation products thereof containing the His-tag. By converting luminescence values to SA concentration using a SA calibration series, the results showed that incubation of chorismate with the combination of the recombinant proteins EntC and His-PRXR1 resulted in enhanced levels of SA, in a His-PRXR1-dependent manner (Fig. 6b). Because isochorismate also spontaneously converted to salicylate (DeClue et al. 2006; Luo et al. 2011), basal amounts of SA were also detected with the combination of chorismate and EntC. Without EntC, incubation of PRXR1 with chorismate also resulted in a slightly higher SA content, possibly because *Acinetobacter* is likely to contain an *ICS* gene that could provide low amounts of isochorismate. In the absence of chorismate, PRXR1 alone or with EntC did not produce a SA-related signal. The results indicate that PRXR1 might have IPL activity.

## Discussion

SA is an important plant signaling molecule for defense against biotrophic pathogens and is regulating certain developmental processes. In Arabidopsis, the majority of SA is produced via an ICS-mediated pathway. This pathway involves the conversion of chorismate to isochorismate by the enzyme ICS and isochorismate to SA by the enzyme IPL. ICS has been isolated and has been well-studied. However, evidence for IPL activity in Arabidopsis is still lacking. In this article, we describe an *E. coli* biosensor that can be used to screen cDNA expression libraries for finding genes encoding proteins with IPL activity. The in vivo screening resulted in the identification of PRXR1, which belongs to the peroxidase family. Increased production of SA from chorismate (in the presence of EntC and PRXR1) in vitro suggests that PRXR1 could indeed have IPL activity.

In this study, we designed a new *E. coli* SA biosensor instead of the previously described *Acinetobacter* ADPWH_*lux* to screen the Arabidopsis cDNA library. This was necessary because plant protein expression systems for *A*. ADPWH_*lux* do not exist. The biosensor module was obtained by PCR from *Acinetobacter* ADPWH_*lux* genomic DNA and consisted of the SA-responsive P*salA* promoter in front of the *lux* operon and the constitutively expressed gene *salR* encoding the regulator protein. These modules were cloned in an *E. coli* plasmid vector, which after transformation resulted in a functional *E. coli* biosensor for detection of either exogenously supplied SA or intracellularly generated SA.

Our biosensor XL1-LUX was responsive to SA concentrations in the low µM range (above 2 µM on solid SA supplied medium), as indicated by detectable luminescence (Fig. 1). It was surprising that the optimum temperature for dynamically detecting a salicylic acid concentration range was found to be 28 °C or lower, which is lower than the optimum growth temperature for *E. coli* and most *Acinetobacter* strains. Apart from being an activator of the *salA* promoter in the presence of SA, SalR was found to be a repressor of the *salA* operon in the absence of SA (Zhang et al. 2012). We speculate that SalR represses *salA* gene expression by negative regulation via the binding site (TTCA-N_12_-TGAT) in the promoter of *salA* in *Acinetobacter* sp. ADP1 (Schell 1993). The induction of *luxCDABE* by SalR at 37 °C in the absence of SA suggests that the conformational change that turns SalR from repressor to activator is subject to temperature in *E. coli*. Compared to when a gene is integrated in the chromosome, the copy number when the gene is present on a plasmid is much higher, which may also account for leaky expression. Moreover, it is possible that in the reconstructed operon, the P*salA* promoter functions sub-optimally, or that *salR* gene expression is lower in *E. coli* than in *Acinetobacter*. This would explain our finding that the expression of *luxCDABE* is activated less efficiently by SalR in *E. coli* than in *Acinetobacter*, and that *E. coli* cells show some leaky expression under non-induced conditions compared to *Acinetobacter* at 37 °C. To improve the sensitivity response to SA and decrease the leaky expression of *lux*, the intergenic region between the SalR binding site and the *luxCDABE* gene could be modified, for instance, by substitution of an *E. coli* compatible ribosome binding site, or by altering the SalR binding affinity of P*salA* (Park et al. 2005; Blazeck and Alper 2013; Jha et al. 2014).

The sensor was tested for detection of endogenously produced SA after transformation with a plasmid harboring the *PmsB* gene encoding bacterial IPL. Expression of the *lux* operon was observed after induction with IPTG, but low-level leaky expression of luciferase activity was noticeable in uninduced cells (Fig. 2). We transformed the biosensor with a cDNA plasmid library of TCV-infected Arabidopsis to screen for luminescent clones (Figs. 3, 4). We identified 12 clones that were in frame with the β-galactosidase α-fragment and that were able to activate *lux* gene expression in *E. coli*, and which we therefore considered to express IPL candidates (Table S1). Subsequent analyses confirmed that at least one, PRXR1, might have IPL enzyme activity (Figs. 5, 6).

**Fig. 7:**
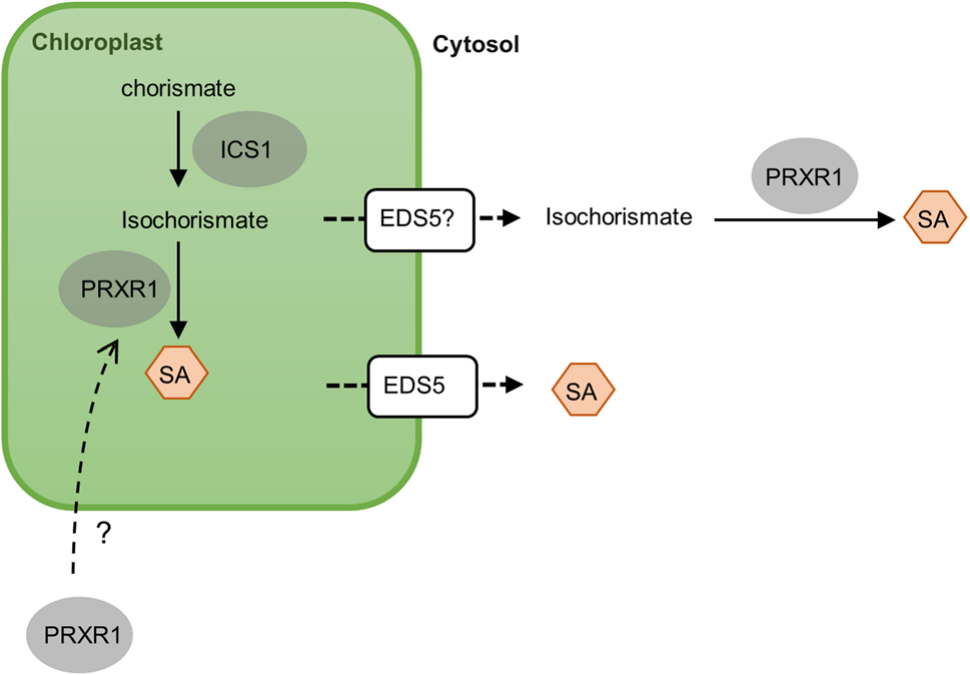
Proposed model describing the isochorismate-dependent SA biosynthesis pathway in Arabidopsis. ICS1 converts chorismate into isochorismate. Isochorismate is exported from the chloroplasts to the cytosol by EDS5 or other transporters (indicated by question mark) and converted into SA by PRXR1. Alternatively, the formation of SA is in the chloroplasts. In this case, PRXR1 is re-localized into the chloroplasts and functions as IPL

The isochorismate pathway is at least partly (ICS, EDS5) localized in the chloroplast, which suggests that the hypothetical IPL could also be chloroplast localized. Previous findings by Chong et al. (2015) suggested that PRXR1 interacted with an endoplasmic reticulum (ER)-localized heat-shock protein 90.7 (HSP90.7), which indicates that PRXR1 is possibly localized in the ER. PRXR1 belongs to the group of secretory class III peroxidases (EC 1.11.1.7) only found in plants. These are heme-containing glycoproteins able to oxidize various substrates, utilizing hydrogen peroxide as electron donor. Identified substrates include phenolic compounds, auxin, and secondary metabolites (Welinder et al. 1992; Ruiz-Duenas et al. 2001; Passardi et al. 2004). Together, these results lead to the following model (Fig. 7). We speculate that isochorismate is transported from the chloroplasts to the cytosol by the MATE transporter EDS5, which is essential for accumulation of cytoplasmic SA (Nawrath et al. 2002; Serrano et al. 2013; Yamasaki et al. 2013). Once isochorismate is in the cytosol, PRXR1 may catalyze the conversion of isochorismate to SA. Alternatively, PRXR1 may be re-localized into the chloroplasts to convert isochorismate into SA.

We showed that only a small amount of chorismate was transformed into SA via the combination of EntC and PRXR1, suggesting that the protein assay conditions were not optimal. Alternatively, PRXR1 may play only a minor role in SA biosynthesis, and other enzymes, either chloroplast or cytoplasm localized, are involved in this process as well. Another possibility relies on the fact that isochorismate spontaneously undergoes rearrangement to form isoprephenate without enzyme catalysis (DeClue et al. 2006; Luo et al. 2011). This could suggest that isoprephenate in the reaction mixture is converted to SA by PRXR1. In addition, slightly enhanced luminescence was observed in the reaction mixtures containing chorismate and EntC only, implying that a small amount of isochorismate spontaneously converted into SA. Therefore, it could also be possible that PRXR1-increased SA production relies on a stabilizing effect of the enzyme on EntC, which would lead to enhanced production of isochorismate and subsequent increased spontaneous conversion to SA.

## Materials and methods

### Reporter plasmid construction

Genomic DNA was isolated from *Acinetobacter* sp. ADPWH_*lux* (Huang et al. 2005) with the genomic DNA purification protocol from Cold Spring Harbor (Green and Sambrook 2012). The *SalA* promoter region, *salR* with promoter and *luxCDABE* were amplified from the *Acinetobacter* sp. ADPWH_*lux* genomic DNA and cloned into pJET1.2 (Thermo Scientific), using primers shown in Table S2. The *salA* promoter digested from pJET1.2 with BamHI and EcoRI, introduced upstream of *salR* in pJET1.2 digested with BamHI and EcoRI to generate plasmid pSALAR. *luxCDABE* was excised from pJET1.2 with EcoRI and inserted between the *salA* promoter and *salR* region in the pSALAR plasmid, resulting in plasmid pLUX, which was transformed into *E. coli* strain XL-1 Blue MRF’. Plasmids were selected on carbenicillin (200 µg/mL) and 500 µM SA. The plates were photographed in the dark after auto-exposure in a Gel Doc XR + Gel Documentation System (Bio-Rad). The reporter plasmid containing the P*salA::luxCDABE:salR* cassette was named pLUX.

### Transformation and validation of luciferase expression

Reporter plasmid pLUX was transformed into *E. coli* XL-1 Blue MRF’ cells and grown on LC agar plates containing carbenicillin (200 µg/mL) at room temperature for 3 days. Individual clones were inoculated onto LC agar plates containing carbenicillin (200 µg/mL) and SA at various concentrations ranging from 0 to 100 µM and incubated at 28 °C for 2–3 days. The luminescence was detected using an image scanner (ProXPRESS 2D Proteomic Imaging System, Perkin-Elmer) or X-ray film (Fuji). Competent *E. coli* XL1-LUX cells were prepared from liquid media containing carbenicillin (200 µg/ml) and grown at room temperature (22 °C) until OD_600_ = 0.4, after which the procedure followed the standard protocol (Inoue et al. 1990). For SA induction of *E. coli* XL1-LUX, 50 µL of cell cultures (OD_600_ = 0.4) were mixed with 20 µL of various SA concentrations (0–500 µM) and 60 µL LC medium, and incubated for 1 h at room temperature (22 °C), 28 and 37 °C, respectively. The luminescence was determined immediately by using a Victor light 1420 luminescence counter (Perkin-Elmer).

### Construction of a double plasmid system for sensing endogenous SA in *E. coli*

*PmsB* with His-tag sequence was amplified from *P. fluorescens*, cloned into pBK-CMV and transformed into *E. coli* XL1-LUX and selected on LC agar plates with carbenicillin (100 µg/mL) and kanamycin (25 µg/mL). Individual clones were selected and inoculated on LC agar plates with carbenicillin (100 µg/mL), kanamycin (25 µg/mL) and 1 mM IPTG. The cells were incubated at room temperature for 3–4 days. Luminescence was detected using X-ray film.

### cDNA library screening in *E. coli*

A cDNA library prepared from a mixture of RNAs from 18 days old Arabidopsis ecotype Di-17 leaves harvested at 6, 12, 19 and 24 h after TCV infection at a ratio of 1:1:1:2 was constructed and amplified using the Stratagene Zap cDNA synthesis and cloning kit and the ZAP Express vector (Cooley et al. 2000). The lambda phage cDNA library was converted into a kanamycin-resistant pBK-CMV plasmid library according to the instruction manual (Stratagene). The cDNA library was transformed into *E. coli* XL1-LUX and grown on LC agar plates containing carbenicillin (100 µg/mL), kanamycin (25 µg/mL) and 1 mM IPTG. The cells were incubated at room temperature for 3–4 days and luminescence was recorded using an image scanner. Luminescent clones were streaked onto fresh selected plates. Luminescence was captured after growing at room temperature for another 2–3 days. Plasmids (pBK-CMV and pLUX) were extracted from the *E. coli* colonies with relatively high luminescence and transformed into *E. coli* XL1-Blue MRF’. The transformants were allowed to grow on LC plates containing carbenicillin (100 µg/mL), kanamycin (25 µg/mL) and 1 mM IPTG at 28 °C for 1 day, followed by 2–3 days at room temperature. Subsequently, luminescence was captured using an image scanner.

Single plasmids were isolated from *E. coli* and sequenced. Sequenced plasmids were transformed into *E. coli* BL21 (DE3) pLysS. The cells were grown in LC medium containing kanamycin (50 µg/mL) and chloramphenicol (50 µg/mL) at 37 °C until OD_600_ = 0.6, induced with 1 mM IPTG and grown overnight at 18 °C. The cells were collected by centrifugation, resuspended in reaction buffer (100 mM Tris–HCl pH 7.0, 15 mM MgCl_2_, 1 mM DTT, 5% glycerol) and sonicated. Crude soluble cell extracts were obtained after centrifugation. Reaction mixtures (500 µM chorismate, 10 µg EntC purified protein, 50 µL cell extract) were incubated at 30 °C for 2 h. Twenty microliters supernatant from the reaction mixtures were mixed with *Acinetobacter* sp. ADPWH_*lux* cell culture (OD_600_ = 0.4) and 50 µL LC medium, incubated at 30 °C for 1 h. The luminescence was measured using a luminescence counter.

### Expression and isolation of recombinant protein

The full-length coding sequence of PRXR1 was amplified from Arabidopsis cDNA and cloned into pASK-IBA45plus. Recombinant protein was expressed in *E. coli* strain BL21 (DE3) pLysS and purified with Ni–NTA Agarose (Qiagen) according to the manufacturer’s protocol. EntC purification was performed as previous described (Zhou 2018).

Purified protein was dialyzed against buffer containing 100 mM Tris–HCl pH 7.0, 15 mM MgCl_2_, 1 mM DTT, 5% glycerol and stored at − 80 °C. The subunit molecular mass of AtPRXR1 was estimated using 15% SDS-PAGE. The primers used to amplify AtPRXR1 can be found in Table S2.

### Determination of IPL activity

The IPL reaction was measured by coupling with excess isochorismate synthase (recombinant EntC). In brief, the reaction mixture (500 µM chorismate, 16 µg EntC, 3.6–48 µg PRXR1, 100 mM Tris–HCl pH 7.0, 15 mM MgCl_2_, 1 mM DTT, 5% glycerol) in a final volume of 150 µL was incubated for 2 h at 30 °C, vortexed and centrifuged. The supernatant was mixed with *Acinetobacter* sp. ADPWH_*lux* cell culture (OD_600_ = 0.4) and 50 µL LC medium, followed by incubation at 30 °C for 1 h. The luminescence was measured using a luminescence counter.

## Electronic supplementary material

Below is the link to the electronic supplementary material.

Supplementary material 1 (XLSX 10 KB)

Supplementary material 2 (XLSX 9 KB)
